# Real-time Inference and Detection of Disruptive EEG Networks for Epileptic Seizures

**DOI:** 10.1038/s41598-020-65401-6

**Published:** 2020-05-26

**Authors:** Walter Bomela, Shuo Wang, Chun-An Chou, Jr-Shin Li

**Affiliations:** 1 Department of Electrical and Systems Engineering, Washington University in St. Louis, St. Louis, MO 63130 USA; 2 Department of Mechanical & Aerospace Engineering, University of Texas at Arlington, Arlington, TX 76010 USA; 3 Department of Mechanical and Industrial Engineering, Northeastern University, Boston, MA 02115 USA

**Keywords:** Network topology, Data processing, Biomedical engineering

## Abstract

Recent studies in brain science and neurological medicine paid a particular attention to develop machine learning-based techniques for the detection and prediction of epileptic seizures with electroencephalogram (EEG). As a noninvasive monitoring method to record brain electrical activities, EEG has been widely used for capturing the underlying dynamics of disruptive neuronal responses across the brain in real-time to provide clinical guidance in support of epileptic seizure treatments in practice. In this study, we introduce a novel dynamic learning method that first infers a time-varying network constituted by multivariate EEG signals, which represents the overall dynamics of the brain network, and subsequently quantifies its topological property using graph theory. We demonstrate the efficacy of our learning method to detect relatively strong synchronization (characterized by the algebraic connectivity metric) caused by abnormal neuronal firing during a seizure onset. The computational results for a realistic scalp EEG database show a detection rate of 93.6% and a false positive rate of 0.16 per hour (FP/h); furthermore, our method observes potential pre-seizure phenomena in some cases.

## Introduction

The human brain is undoubtedly one of the most complex dynamic systems known to mankind, which consists of billions of interconnected neurons^1^. The connectivity patterns across different brain regions could convey evidences for advancing and supporting the understanding of cognitive and behavioral functions^2–5^. In the past decades, many research studies have been devoted to discovering the process and mechanisms that govern the communication between neurons; in particular, how and why neuronal networks are formed for various neurological disorders. Epilepsy is one of the most common central nervous system disorders, following Alzheimer’s disease and stroke. According to recent statistics^6^, nearly 4% of people across different ages are diagnosed with epilepsy and suffer from epileptic seizure occurrence and recurrence during their lifetimes. Epileptic seizures develop with a sudden abnormal surge of electrical activities of pathological, synchronous neuronal firing in all brain regions or some parts. Seizures can be controlled with medications and/or invasive surgeries in 70% of diagnosed patients^7^. However, prompt detection or even prediction of seizure onsets remains a critical and challenging task to date because of its high variation patterns of occurrence and manifestation from one individual to another. To this end, a lot of efforts have been given to developing subject-specific, data-driven methodologies aiming at ultimately improving individualized seizure onset detection and prediction^8^. Electroencephalography (EEG) recordings in high temporal resolution, regardless of arguable evidence of poor spatial resolution and low signal-to-noise ratio (SNR), are useful amongst neuroimaging techniques allowing clinicians to collect and monitor global neuronal activity information in the brain of epileptic patients.

Epileptic EEG signals are in the form of multiple non-linear, non-stationary time series and represent the underlying dynamics of the brain system and interactions among neurons. To monitor and detect abnormal and excessive firing during seizure onsets, there have been a variety of data analysis techniques proposed for extracting informative biomarkers or connectivity patterns from EEG signals, including linear time-frequency analysis (e.g., Fourier transformation, wavelet transformation)^9–11^ and non-linear methods (e.g., Lyapunov exponents and entropy)^12–14^. Moreover, for seizure detection or prediction, in practice, the above-mentioned analysis methods are usually developed and integrated within a machine learning framework^7,15,16^. In many of these previous studies, EEG signals were processed to extract features (or biomarkers) in individual channels, and predictive models were then developed and trained with a large number of extracted features using state-of-the-art machine learning techniques (e.g., support vector machine, logistic regression, neural nets). The results generated by these methods promised a significant improvement of prediction or detection accuracy for various benchmark databases^17–19^.

However, many methods in the literature did not account for synchronous neuronal interactions across the brain^20^, and also were unable to characterize the dynamic nature of non-linearity and non-stationarity properly^21^. To this end, nonlinear dynamic learning approaches were proposed to present the epileptic brain as a dynamic network and investigate its spatiotemporal synchronization properties^12,22,23^. The underlying dynamics of global or local epilepsy can be assessed through the change of spatiotemporal synchronization (or dynamic network) patterns^24,25^. Some studies have shown great promises in seizure onset detection and prediction with network-based features, e.g., degree, shortest path, clustering coefficients, and the algebraic connectivity (Fiedler eigenvalue^26^), characterizing the network property^27–29^.

Still, accurately capturing the time-varying dynamics of interconnected neurons (recorded by EEG signals) remains a challenging question due to the difficulty to infer networks from low signal-to-noise ratio data in a more effective way. Pioneering work on automatic recognition of epileptic seizures has demonstrated that it is possible to detect seizures by decomposing the EEG signals into elementary waves and analyzing rhythmic activities in the frequency band 3 to 20Hz^30^. Subsequently, a variety of graph-theoretic based approaches were proposed to construct the epileptic brain network using different techniques. For instance, some researchers computed the correlation^31,32^ between EEG signals pair-wise and determined the existence of connectivity between channels by thresholding the coefficient values^32,33^, while others used a phase lag index which was computed using the angles obtained from the Hilbert transform of the EEG signals^33^.

In this study, we propose a new computational method that processes EEG signals in real-time, which in turn can be used for automated seizure control treatments in clinical practice. We utilize a technique for inferring the connectivity of networks (ICON)^34^, which reveals synchronization patterns during seizure onsets by estimating the coupling functions between EEG channels. The seizure onset is detected on a basis of the synchronization property of the recovered time-varying network inferred from multi-channel EEG data. To accelerate the seizure detection algorithm, we introduce a more computationally efficient approach derived from the ICON method and infer the time-varying network by computing the power of the coupling functions using Fourier transform (FT) applied on short epochs (0.25–1 second) of multi-channel EEG data (referred as *FT method*). Several methods in the literature have used the discrete wavelet transform (DWT)^35,36^ in place of the Fourier transform that is considered to suffer from large noise sensitivity, but by combining the power and phase spectrum to infer the epileptic brain network, our approach significantly improves the robustness of the detection algorithm that effectively rejects artifacts. Furthermore, machine learning (ML) techniques are often used for seizure detection, and in the literature, various features are used as input to the ML model, e.g., the energy in EEG signals computed using wavelet transforms^17^. However, the performance of the ML models depends in part on the goodness of the feature(s) used. It is then expected that our graph-theoretic approach, which offers an excellent noise reject ratio, will improve the performance of seizure detectors.

## Results

**Figure 1:**
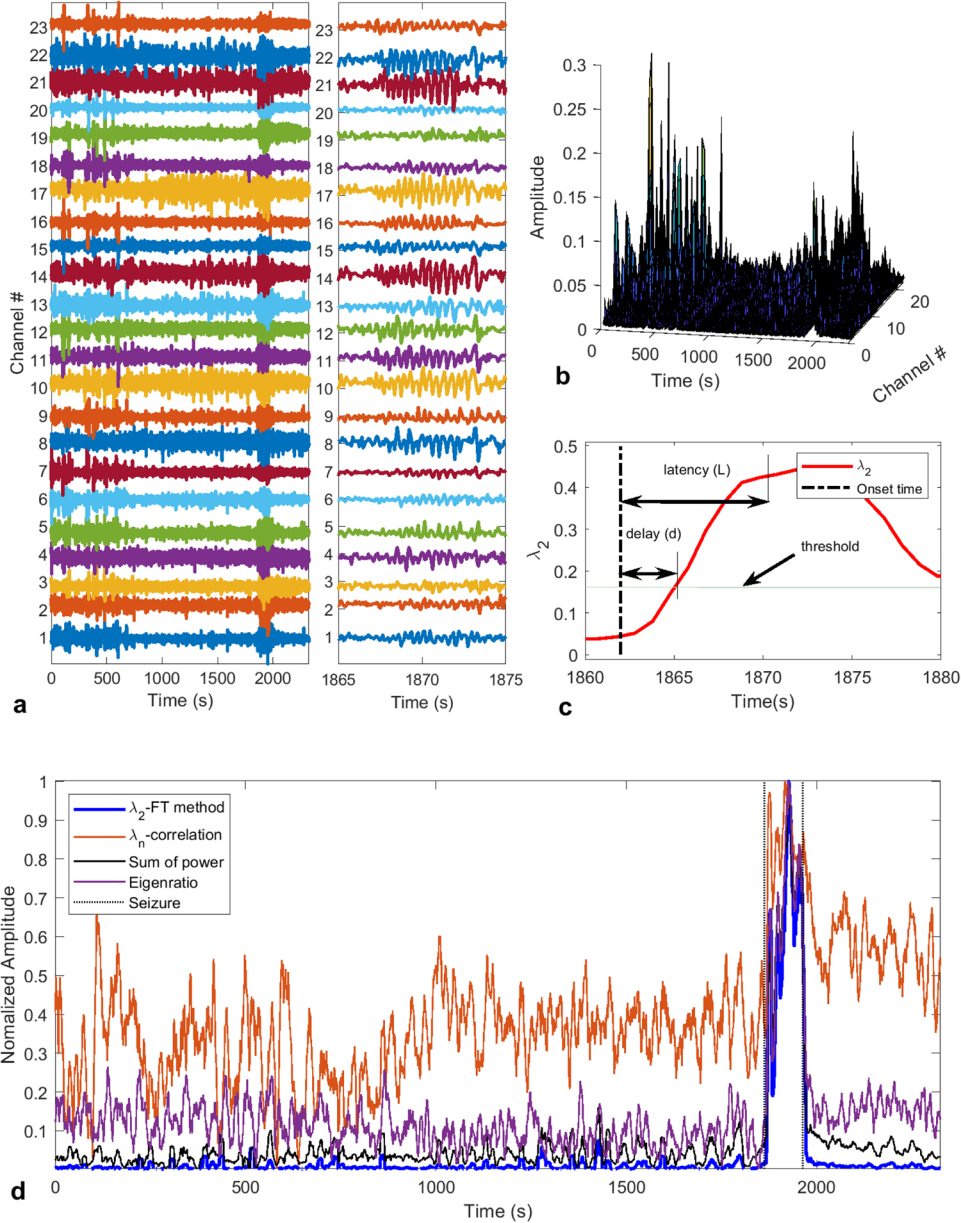
Illustration of the seizure detection process. (**a**) Sample EEG signals (23 channels) showing a seizure occurring around time = 1870s. The left panel shows the entire recorded session with some artifacts at the beginning. The right panel shows a 10s segment with pre-seizure and seizure activities. (**b**) 3-D plot of the power spectrum of EEG channels showing strong power surges in some channels due to artifacts before time = 1000s, and some of them are stronger than the surge of power due to seizure. (**c**) Depiction of how the delay and latency are measured from the recorded seizure onset time with $L=d+6s$. (**d**) Fiedler eigenvalue of the synchronization network denoted *λ*_2_-FT method. Seizure occurrence is characterized by an increase of *λ*_2_ and the seizure interval is delimited by the dotted lines. The other features are the *λ*_*n*_ obtained from the correlation matrix (where the diagonal entries have been set to zero to avoid self-coupling), the sum of the power spectrum obtained with FFT and the eigenratio ${\lambda }_{2}/{\lambda }_{n}$ obtained from FT-method, respectively.

It is believed that epileptic seizures are characterized by widespread synchronous firing of neurons, and this synchronization often appears in a certain frequency band^33,37^, e.g., theta band (4–8 Hz)^38,39^. Hence, monitoring the brain electrical activity using EEG and analyzing its spectral information in the frequency domain can reveal seizure occurrences. However, EEG signals are often corrupted by noise, artifacts and/or brain activities (see Fig. 1a) that can be confused with seizure activities, and this can render accurate detection of seizure onsets very difficult. It is common practice to use either low-pass or band-pass filters to remove unwanted frequency components from EEG signals (see the right panel of Fig. 1a for a ten-second segment of non-seizure followed by seizure activities, respectively) before proceeding to its analysis^40–42^. The FT method presented herein, however, eliminates the need to use any such pre-processing of data.

### FT method

The performance of the proposed FT method was evaluated on the pediatric scalp EEG data from the CHB-MIT database available on the PhysioNet website^43^. The database contains 24 cases collected from 23 subjects. Note that in this paper we refer to each case by a patient number (from #1 to #24). We employed a graph-theoretic approach to fuse the information contained in the 23 EEG channels. This resulted in a better noise and artifacts rejection, even though short-time Fourier transform (STFT) was used (see Fig. 1). Indeed, one can see from Fig. 1b that there are significant peaks in the power spectrum in the time interval of 0 to 700 seconds, however, these power surges do not correspond to any seizure activity. Furthermore, from the second-smallest eigenvalue, *λ*_2_, of the Laplacian matrix of the inferred network, one can clearly distinguish the noise and artifacts from seizure activities (Fig. 1d). The Fiedler eigenvalue also defines the algebraic connectivity of a graph; in other words, it characterizes the graph synchronizability.

We tested our method on 17 patients, and excluded patients that had seizures characterized by amplitude depression (AD), and the results are summarized in Table 1. Overall, we achieved a sensitivity of 93.6% with a false detection rate of 0.16 FPs/h. The average seizure detection time was 16 seconds from the indicated seizure onset time in the dataset (this was obtained by considering a 6 second decision time). A closer look at the EEG signals, for those patients with high detection delay or latency, revealed that it was due, in most cases, to seizures starting with AD before the occurrence of synchronization that is characterized by coherent high amplitude oscillations. Since this algorithm is not designed to detect amplitude depression (because the detection is based on *λ*_2_ going above a threshold), for these cases, we observed large latencies.

**Table 1:** Results summary for the FT method.

Patient #	NS	TP	FP	L (s)	S (%)	D (s)	FP/h
1	7	7	3	8.57	100.00	2.57	0.07
2	3	2	7	12.50	66.67	6.50	0.20
3	7	7	9	7.43	100.00	1.43	0.26
4	4	2	5	36.50	50.00	30.50	0.14
5	5	5	0	11.40	100.00	5.40	0.00
7	3	3	3	18.67	100.00	12.67	0.05
8	5	5	0	13.60	100.00	7.60	0.00
9	4	4	3	9.25	100.00	3.25	0.04
10	7	7	2	9.86	100.00	3.86	0.04
11	3	1	0	30.00	33.33	24.00	0.00
17	3	3	36	22.33	100.00	16.33	1.80
18	6	5	6	11.20	83.33	5.20	0.17
19	3	3	0	33.67	100.00	27.67	0.00
20	8	8	8	14.25	100.00	8.25	0.29
22	3	3	3	18.33	100.00	12.33	0.10
23	7	7	5	3.14	100.00	−2.86	0.19
24	16	16	9	12.31	100.00	6.31	0.42
Total (17 patients)	94	88	99				
Mean				16.06		10.06	
Sensitivity		93.62%					
FP/h			0.16				

NS: Number of seizures; TP: True positive; FP: False positive; L: Latency; S: Sensitivity; D: Delay; FP/h: False positive per hour.

The FT method presented here achieves comparable performance for seizure onset detection with a lower false detection rate than most algorithms proposed in the literature^16,42,44^. However, our method presents some computational advantages. First, by using a short sliding window of size between 0.25 to 1 second, the algorithm requires less memory storage and has a very short runtime, hence making it a good candidate for implementation on portable devices for real-time seizure detection. The second advantage is that we achieved a very good performance with only one extracted feature (*λ*_2_); however, extracting more features from the inferred graph, and using more sophisticated machine learning algorithms can significantly improve the sensitivity and the false detection rate. Note that the results presented here were obtained without using any machine learning algorithms, and instead a simple thresholding technique was used as shown in Fig. 1c.

### ICON method

**Figure 2:**
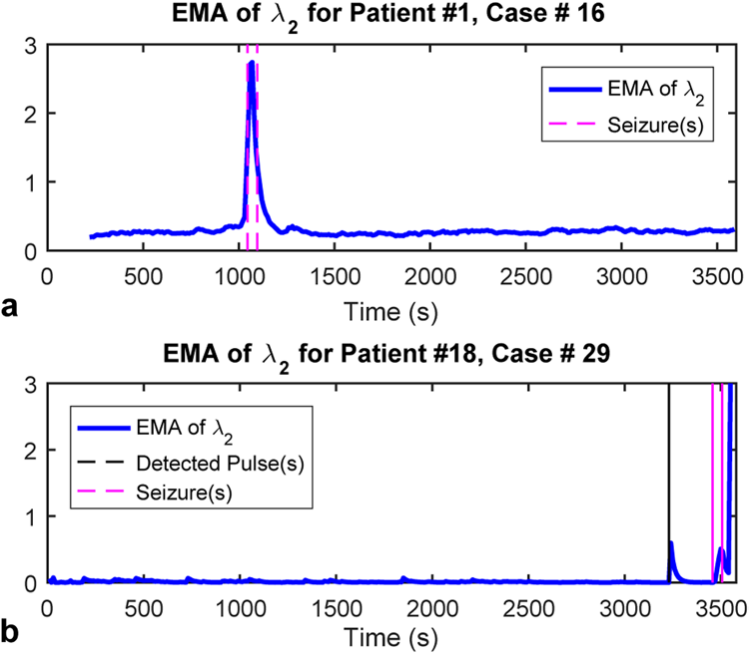
Examples of the results using the ICON method. (**a**) For case #16 of patient #1, the changes of the exponential moving average (EMA) of *λ*_2_ of the recovered network’s Laplacian matrix (blue) matches the recorded seizure onset and offset times (magenta). (**b**) For the case #29 of patient #18, besides for the changes of EMA indicating the seizure onset and offset times (magenta), there was a peak (black) 227 seconds before the seizure actual onset time.

The performance of the ICON method was evaluated using the same dataset. Figure 2 shows two examples, case #16 for patient #1 and case #29 for patient #18, where ICON captured seizure onset through the abnormalities in synchronizability of the constructed dynamic brain networks. The synchronizability^45^, given by the exponential moving average (EMA) of *λ*_2_^45,46^, can be derived directly from the dynamic interactions between brain regions inferred by ICON. Figure 2a illustrates a case (for patient #1, case #16) where ICON accurately captured seizure onset and offset times as recorded in the dataset, whereas Fig. 2b shows a case (for patient #18, case #29) of pre-seizure event that was detected 227 seconds before the actual seizure onset.

**Figure 3:**
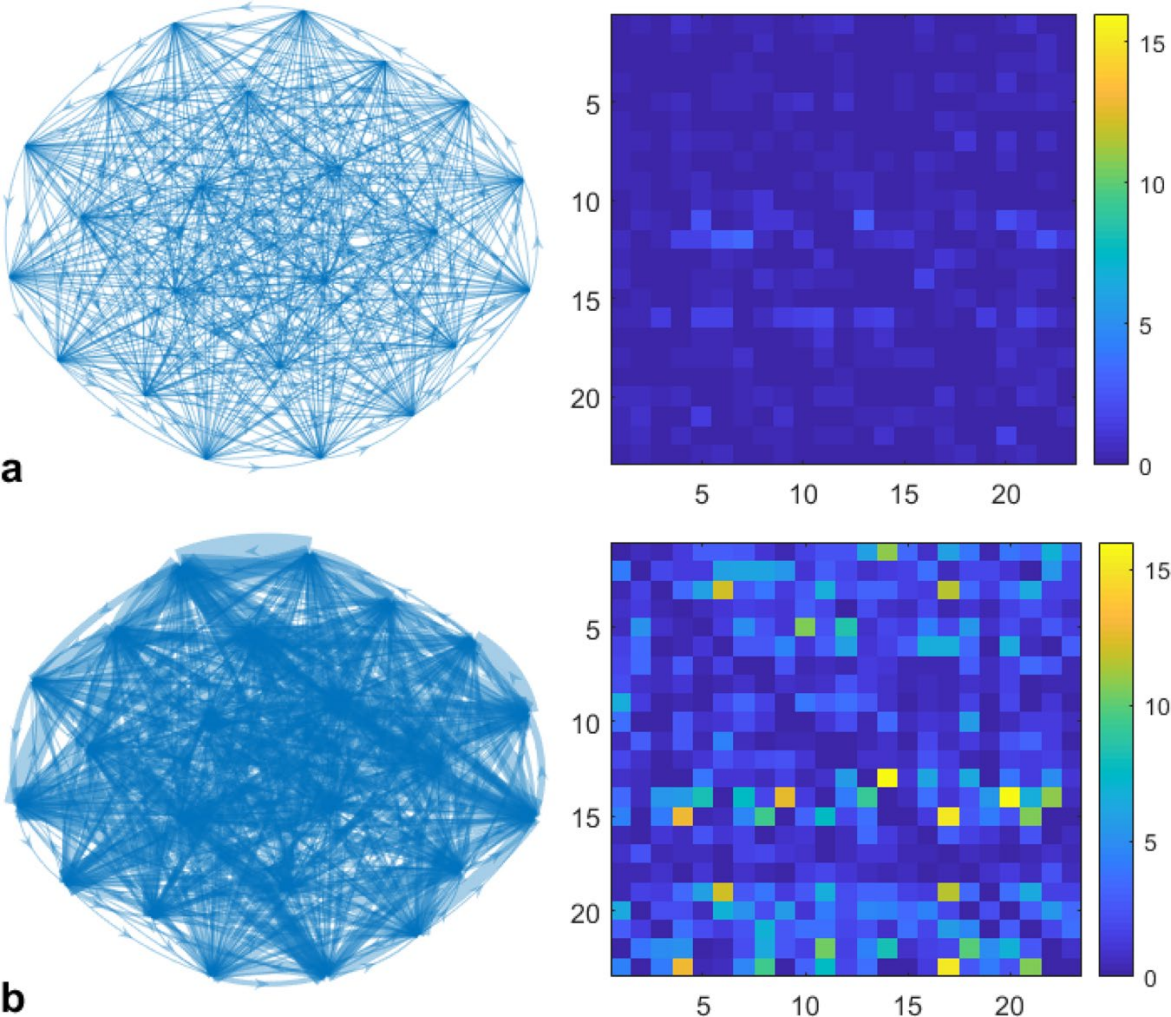
Networks (graph representation and the heat-map representation of the corresponding adjacency matrix) of the normal state (**a**) and seizure state (**b**).

Moreover, Fig. 3 depicts the differences between the brain networks during normal brain activity (Fig. 3a) and seizure occurrence (Fig. 3b), inferred with ICON for the case presented in Fig. 2b. We can see that during a seizure, the strength of interactions between vertices, represented by weighted edges in Fig. 3b significantly increases. The summary of the results for all of the 24 patients is given in Table 2. Overall, a sensitivity of 78.79% was achieved, with a false detection rate of 0.02 FPs/h. However, we can achieve a sensitivity of 89.36% with a false detection rate of 0.01 FPs/h for only 17 patients considered for the FT method. The average seizure detection time was 4 seconds from the indicated seizure onset time and possible pre-seizure phenomena were observed for some patients (#11-#13, #15, and #18-#24).

**Table 2:** Results summary for the ICON method.

Patient #	NS	TP	FP	L (s)	S (%)	D (s)	FP/h
1	7	7	0	2.00	100.00	−4.00	0.00
2	3	1	0	0.00	33.33	−25.00	0.00
3	7	7	1	5.14	100.00	−0.86	0.03
4	4	4	0	10.25	100.00	4.25	0.00
5	5	5	0	18.80	100.00	12.80	0.00
6	10	1	0	80.00	10.00	74.00	0.00
7	3	3	0	11.00	100.00	5.00	0.00
8	5	4	0	0.50	80.00	−5.50	0.00
9	4	4	0	0.25	100.00	−5.75	0.00
10	7	5	0	1.60	71.43	−4.40	0.00
11	3	2	0	8.00	66.67	2.00	0.00
12	40	32	2	3.25	80.00	−2.75	0.08
13	12	10	5	11.40	83.33	5.40	0.15
14	8	3	0	0.00	37.50	−6.00	0.00
15	20	17	2	2.41	85.00	−3.59	0.05
16	10	7	0	5.57	70.00	−0.42	0.00
17	3	2	1	0.00	66.67	−6.00	0.05
18	6	5	3	4.00	83.33	−2.00	0.08
19	3	3	0	0.00	100.00	−6.00	0.00
20	8	7	1	2.71	87.50	−3.28	0.04
21	4	2	0	0.00	50.00	−6.00	0.00
22	3	3	0	0.00	100.00	−6.00	0.00
23	7	7	1	1.71	100.00	−4.28	0.04
24	16	15	0	1.20	93.75	−4.80	0.00
Total (24 patients)	198	156	16				
Mean				4.44		−1.68	
Sensitivity		78.79%					
FP/h			0.0186				

NS: Number of seizures; TP: True positive; FP: False positive; L: Latency; S: Sensitivity; D: Delay; FP/h: False positive per hour

## Discussion

In this work, we provided a data-driven, dynmaic learning approach for seizure detection that was tested on the widely used benchmark CHB-MIT scalp EEG database^43^. The ICON method, which constructs a dynamic brain network to infer its connectivity from noisy measurement data, establishes a dynamic graph-theoretic approach to inferring the network dynamic topology and hence detecting the seizure onset and offset times by capturing the network’s abnormalities. This work connects the brain network properties, such as connectivity and synchronizability^37^, with the occurrence of seizures. The hypothesis that there is a certain level of synchrony in brain signals during seizure led to a technique for inferring the epileptic brain network using mean phase coherence^47^. This approach was used to analyze changes to graph centralities to shed more light on the role of constituents of evolving epileptic networks that recurrently transit into and out of seizures^48^. Furthermore, fundamental questions such as which nodes are connected by a predictive edge and which network modifications constitute a pre-seizure state were explored^48^. However, with ICON we mainly explore the seizure predictive capability of the second eigenvalue which is a measure of network algebraic connectivity.

This method requires no prior information for data processing, and hence it can be applied to the entire dataset.

As seen in Table 2, the ICON method is sensitive to any network’s abnormalities, which in turn guarantees the small latency for detecting seizure onset and offset times. Furthermore, since it can capture abnormality in the network dynamic topology, which also includes the one caused by neuronal disorders that might trigger the occurrence of seizures, this method can capture and reveal the patient-specific pre-seizure phenomena (See Fig. 2b). The challenge, however, is then how to relate these abnormalities to seizures while differentiating them from noise and artifacts, for example. This task will be difficult to achieve with the thresholding technique used in this paper, but using an appropriate machine learning model (e.g., a neural network) with multiple features extracted from the inferred network has the potential to uncover those hidden patterns that were difficult to detect, and can improve the detector accuracy or even predict seizure occurrence.

In general, the ICON method provides an innovative angle, through constructing a complex dynamic brain network, to detect epileptic seizures. This bridges the occurrence of seizures with its cause and effects, as shown in Fig. 3, which in turn enables future research studies on treating neuronal disorders like epileptic seizures.

While our ICON method can detect different types of seizures from the recovered network properties and hence, was tested on the entire dataset, the FT method cannot detect seizures that are characterized by AD. Therefore, patients #06, #12, #13, #14, #15, and #16 were excluded from the evaluation presented in Table 1. Although this method is not capable of detecting most seizure onsets for these patients, the false alarm rate is still very low. The graph-theoretic approach adopted here performed comparably well to some algorithms proposed in the literature (see Table 3). It is important to note that we achieved good performances without using sophisticated machine learning techniques and without additional features. There are other methods that are also suitable for real-time applications, e.g., the approach based on the phase-slope index of directed influence applied to multichannel electrocorticogram (ECoG) data^49^ and which uses a threshold to assess seizures. In the paper by Rana *et al*., they used a more elaborate method for choosing the threshold based on the moving average of recent activity to accommodate variability between patients and slow changes within each patient over time^49^. Furthermore, by an adequate choice of parameters, they were able to achieve high sensitivity. In our work, on the other hand, we chose a constant threshold for each patient, and we are confident that with our approach, if we use a more elaborate technique for selecting a threshold or if we extract more features from the inferred synchronization network and combined with a good machine learning technique, we can improve the performance of the seizure detector. Recently, a paper has shown that using multiple features with a machine learning model (e.g., a neural network) improves the accuracy of the seizure detector^50^.

**Table 3:** Previous Results based on the same dataset.

	Sensitivity	FP/h	Latency (s)
Shoeb^51^	96.00%	0.21	4.60
Ahammad *et al*.^17^	98.5%	14.4%*	1.76
Thodoroff *et al*.^7^	85%	0.80	Not Reported
Samiee *et al*.^19^	70.19%	2.26%*	Not Reported
Zabihi *et al*.^54^	88.27%	6.79%*	Not Reported
Bhattacharyya and Pachori^10^	97.91%	0.43%*	Not Reported
Khanmohammadi and Chou^18^	96.00%	0.12	4.21
Fan and Chou^46^	~97%	8.61%	6–7
Akbarian and Erfanian^50^	98.67%	~1%	Not Reported

*In these studies, the false positive rate is reported as a percentage of the number of misclassified seizure-free epochs.

While the FT method can be used in automated real-time seizure control systems, the ICON method can help physicians better study pre-seizure phenomena. Indeed, this approach was able to identify brain activities that preceded seizure onsets as marked by specialists in the dataset. On average significant changes in the brain activity were observed 1.68 seconds before the actual seizure onset. The main advantage of the dynamic graph-theoretic approach is its ability to reject noise and artifacts (see Fig. 1d), which allowed us to use a minimal number of features and yet, still achieve good performance of the detector. In particular, in Fig. 1d we show various features that have been obtained with other approaches such as the sum of spectral powers and the correlation method. The main observation is that our method generated a feature (*λ*_2_) that is much smoother than the sum of spectral powers and *λ*_*n*_ obtained with the correlation method. We also compared *λ*_2_ to the eigenratio R = *λ*_2_/*λ*_*n*_, however, R was not as smooth as *λ*_2_ as one can see in Fig. 1d, and for some other patients in the same dataset it was worse than shown in Fig. 1d, hence we do not recommend using it as a feature.

## Methods

### Data acquisition and processing

CHB-MIT scalp EEG dataset^43^ is used for the validation of our proposed method, which consists of multi-variate EEG recordings from 23 epileptic patients at Boston Children’s Hospital (5 males of age from 3 to 22; 17 females of age from 1.5 to 19). The data collection followed a protocol approved by the Committee on Clinical Investigations at the Beth Israel Deaconess Medical Center (BIDMC), Boston, Massachusetts, USA, and the Massachusetts Institute of Technology (MIT), Cambridge, Massachusetts, USA^51^. All procedures were performed in accordance with the relevant regulations and guidelines, and all informed consents were obtained before admission to BIDMC General Clinical Research Center took place. The EEG signals were recorded during many days using the International 10–20 system of 23 electrodes with a sampling rate of 256 Hz, and 182 epileptic epochs were marked by domain experts in a subset of 129 sessions of the entire dataset. Note that we only used the sessions with seizure onset epochs. After baseline removal and normalization, the EEG signals were re-referenced to average and a band-pass filter of 1–50 Hz was applied to reduce noise in the signals. These pre-processing steps were performed using functions in EEGLAB toolbox^52^. For real-time implementation, a non-overlapping sliding window of 1 second length was applied to each session of EEG recordings to split the signals into segments.

### Inferring connections of a brain network (ICON method)

In this paper, we provide a universal framework to diagnose seizure, by estimating a dynamic brain network and then revealing its connectivity from the noisy EEG data using our developed ICON (*inferring connections of networks*) method, which we have previously used to determine the topology of networks of oscillators, arising in electrochemistry, neuronal networks, and groups of mice^34^. Hence, by dynamically monitoring the connectivity of the inferred brain network, we capture abnormal activities such as seizures.

We study the evolution of a dynamic brain network consisting of *N* vertices. The brain activities are recorded using EEG sensors with *N* channels, and the dynamics of the brain regions near each channel is modeled by a state variable *x*_*i*_, where $i=1,\cdots ,N$. The brain activities recorded at each vertex hence follow the dynamic law governed by the brain region’s self-dynamics and the interaction among vertices,

1 $${\dot{x}}_{i}(t)=f({x}_{i})+\sum _{j\ne i}{K}_{ij}({x}_{i},{x}_{j}),\,i=1,\cdots ,\,N,$$

where the vector ${x}_{i}(t)\in {{\mathbb{R}}}^{n}$ denotes the temporal state of brain region *i* at time *t*, the function *f* represents the inherent dynamics for the brain region *i*, and *K*_*ij*_, *i*, $j=1,\,\cdots ,\,N$, is the coupling function between brain regions *i* and *j* (*K*_*ij*_ can be different from *K*_*ji*_). The dynamics, i.e., *f* and *K*_*ij*_, and thus the topology of such a directed brain network is problematic to infer from data because of the inherent nonlinearity within and between brain regions as well as the noisy measurement of the EEG data. However, the dynamic interactions between different regions will provide us insight to explain the brain activities through its first-order derivatives, which is more sensitive to capture the abnormality in this complex brain network and hence result in a lower false detection rate of identifying seizure onsets and/or offsets.

**Figure 4:**
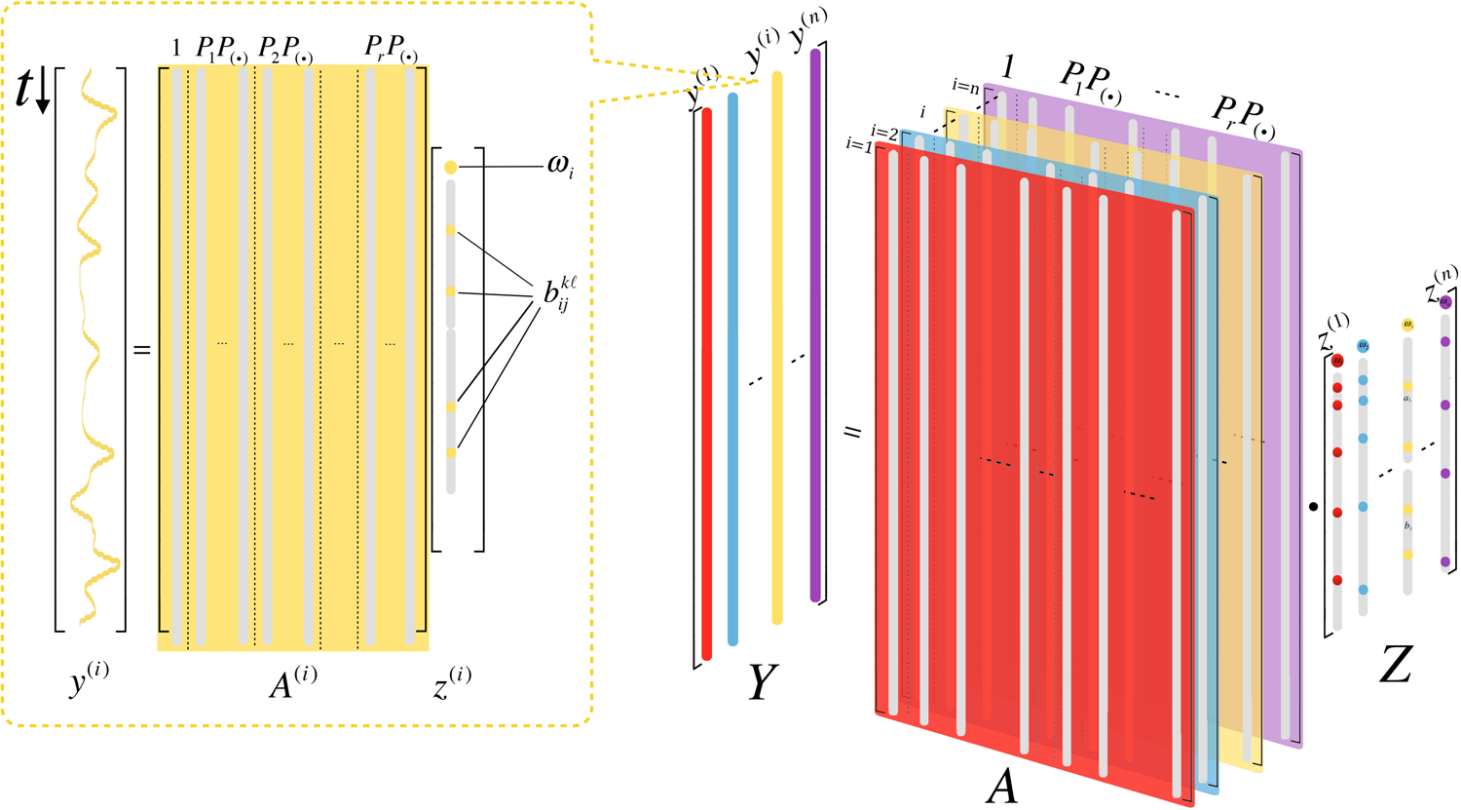
A visualization of the parallel computational architecture for solving the reduced topology estimation problem (3). The right-hand side illustrates the formulation of the problem, where the time-varying interactions ${K}_{ij}$ for each sliding window of the brain region near *i*^th^ channel are estimated in parallel. The inset on the left-hand side describes the linear estimation problem for each channel, where ${{\rm{y}}}^{({\rm{i}})}$ is the data vector, ${{\rm{A}}}^{({\rm{i}})}$ is a matrix involving the 2-dimensional Fourier bases $\{{P}_{k}{P}_{\ell }\}$, and ${{\rm{z}}}^{({\rm{i}})}$ is the coefficient vector to be determined.

The central idea of our approach is to approximate the self and coupling dynamics of each region, *f* and *K*_*ij*_, respectively, by using complete orthogonal bases ${\{{Q}_{k}\}}_{k}$ and ${\{{P}_{k}\}}_{k}$, in particular, the Fourier basis. Then, the dynamical law in Eq. (1) can be expressed as

2 $${\dot{x}}_{i}(t)=\sum _{k}{a}_{k}{Q}_{k}({x}_{i})+\sum _{j\ne i}\sum _{k}\sum _{\ell }{b}_{ij}^{k\ell }{P}_{k}({x}_{i}){P}_{\ell }({x}_{j}),$$

where ${b}_{ij}^{k\ell }$ are the coefficients of the 2-dimensional Fourier basis (with 5 terms) representing the coupling strength ${\alpha }_{ij}$, defined by ${\alpha }_{ij}=\sqrt{\sum _{k}\sum _{l}{({b}_{ij}^{kl})}^{2}}$, that constitutes the Jacobian matrix *J* of the coupling term. Following this strategy based on the orthonormal basis representation and given the EEG data of each agent *i* in the network, the topology estimation can be formulated as a simple linear inverse problem for each agent *i*, given by

3 $$\mathop{\min }\limits_{{z}^{(i)}}\Vert {y}^{(i)}-{A}^{(i)}{z}^{(i)}\Vert ,$$

where *y*^(*i*)^ is the data vector and ${A}^{(i)}\in {{\mathbb{R}}}^{M\times ({r}^{2}N+1)}$ is a matrix composed of the orthonormal bases, in which *M* is the number of data points for each sliding window of channel *i*, and *r* (*r* = 5) is the number of expansion terms in the truncated series; and *z*^(*i*)^ is the coefficient vector that is being determined. Most importantly, this formulation enables independent estimation of the time-varying interactions between brain regions in such a network, so that the numerical computations become efficient (Fig. 4).

With the dynamic network topology revealed by ICON, we can study the synchronization behavior of a given brain network using eigenvalue analysis of the Jacobian matrix $J=[{\alpha }_{ij}]$. Under the assumption that the network connectivity increases when a seizure occurs, the abnormality in the Fiedler eigenvalue, which indicates the synchronizability, would indicate the seizure onset. We achieved the seizure detection by using sliding windows of 10s–20s to guarantee the reliability of the topology recovered by ICON and efficiency of the detection. In this way, ICON can capture the abnormality of the brain network due to seizure efficiently and accurately.

### Inference of brain network from EEG power spectrum (FT method)

For a significant number of patients suffering from epilepsy, seizures appear in EEG data as spiking trains, with frequencies often in specific bands, e.g., 4**–**8 Hz (theta band). However, for some patients’ seizures can appear as amplitude depressions in the brain activity, or sometimes as amplitude death followed by spiking trains. In this paper, we exploit the burst of energy in EEG channels appearing in a given frequency band to form a time-varying synchronization network from which we then extract a single feature (the second eigenvalue, *λ*_2_ of the graph Laplacian matrix). This feature is then compared to a fixed threshold to determine whether a seizure event occurred. The threshold selection was determined on a case by case basis because of the variability of EEG recordings from one patient to another. Furthermore, visual inspection was used to identify the seizure onsets. Various more elaborate methods have been proposed in previous studies, which could be adopted instead of using a simple threshold and visual inpsection, e.g., the method that determines the threshold based on the moving average of previous data points^49^ and neural networks^50^.

Some of the automated seizure detection algorithms currently use Fourier^36^ or wavelet transform^35^ and look at the power spectrum in a certain frequency band^41^ of some predetermined channels. These methods are usually patient dependent in the sense that one needs to know the morphology of the patient’s seizure in addition to the channels that capture the seizure activity. However, it has been shown that algorithms that take into account different seizure morphologies, including amplitude depression, can have good performances and be less patient specific^53^.

In this work, we have presented the ICON method that infers the synchronization network from dynamical EEG measurements. Note that the coupling strengths are computed as ${\alpha }_{ij}=\sqrt{\sum _{k}\sum _{l}{({b}_{ij}^{kl})}^{2}}$in the ICON method, with ${b}_{ij}^{k\ell }$ the coefficients of the orthonormal bases ${P}_{k}{P}_{\ell }$. From Perseval’s theorem, we know that the root mean square (RMS) power of a trigonometric Fourier series of a periodic signal $s(t)={c}_{0}+\mathop{\sum }\limits_{k}^{\infty }{c}_{k}\,\cos (k\omega \,+{\theta }_{k})$ is given by ${P}_{RMS}=\sqrt{\left({c}_{0}^{2},+,\frac{1}{2},\sum _{k}{c}_{k}^{2}\right)}$. Hence, by using the Fourier basis in the ICON method to infer the network coupling strength, we indeed see that the coupling strength between brain regions (vertices) *i* and *j* essentially represents the power of the interaction force between them (power of their interaction function). This analogy to the interaction signal power between vertices inspired a computationally efficient network inference approach, and hence enables online seizure detection.

In the simplified brain network inference approach, we employ the Fourier transform that is applied to the EEG data of each channel *i*. We choose a sliding window of the desired length, e.g., 1 second (or half a second) and compute the power and phase spectrum of each channel using the FFT (Fast Fourier Transform). After obtaining the power magnitudes ${p}_{i}$ (power spectrum) of each channel, we isolate a frequency band, typically between 2–10 Hz (may vary depending on patients), then set the magnitude of each frequency component outside the band [2, 10] Hz, to zero. In order to extract only one feature, and hence suppressing the need to know in advance what channels are measuring seizure activities, we form a synchronization network as follows. Let ${{\mathcal{P}}}_{i}$ be the sum of the squared power spectrum of the *i*^*th*^ channel in a given time interval ${t}_{k}-{t}_{k-1}$ (where *k* is the current epoch). Then the power-related coupling coefficient between two nodes is ${\sigma }_{ij}^{p}={\sigma }_{ji}^{p}={{\mathcal{P}}}_{i}+{{\mathcal{P}}}_{j}$, and the phase-related coupling coefficient is given by ${\sigma }_{ij}^{\phi }=-{\sigma }_{ji}^{\phi }=\frac{1}{N}\mathop{\sum }\limits_{n}^{N}\sin ({\phi }_{j,n}-{\phi }_{i,n})$, where *N* is the number of discrete frequencies in the desired band, e.g., [2, 10] Hz. Hence, the $i{j}^{th}$ entry of the adjacency matrix $A({t}_{k})$ at time ${t}_{k}$ is obtained as ${a}_{ij}=1-\exp (-{\Vert {d}_{ij}\Vert }^{\gamma })$, where ${d}_{ij}={\sigma }_{ij}^{p}\,{\sigma }_{ij}^{\phi }$, with ${d}_{ij}=0$ for $i=j$. Then the Laplacian matrix is computed as $L({t}_{k})=D({t}_{k})-A({t}_{k})$, where $D({t}_{k})$ is the degree matrix. We can then extract a classification feature such as *λ*_2_ at each time step *t*_*k*_ (see Fig. 1d). Note that more features can be extracted from the network and used as inputs to a machine learning model, which we believe will improve the performance of the seizure detector. Note that for the FT method, the results were obtained with the EEG data normalized in the range [−1, 1], and the parameter $\gamma =2$ was used. This parameter can be tuned to increase the robustness of ${\lambda }_{2}$ to noise and artifacts, however, a large value, i.e., $\gamma  > 3$ might reduce seizure detection sensitivity because the value of ${\lambda }_{2}$ might be too small, hence causes some numerical issues.
